# Health service use in families where children enter public care: a nested case control study using the General Practice Research Database

**DOI:** 10.1186/1472-6963-12-65

**Published:** 2012-03-16

**Authors:** Douglas E Simkiss, Nicholas J Spencer, Nigel Stallard, Margaret Thorogood

**Affiliations:** 1 Health Sciences Research Institute, Warwick Medical School, University of Warwick, Coventry CV47AL, UK; 2 School of Health and Social Studies, University of Warwick, Coventry CV4 7AL, UK; 3 Health Sciences Research Institute, Warwick Medical School, University of Warwick, Coventry CV47AL, UK; 4 Health Sciences Research Institute, Warwick Medical School, University of Warwick, Coventry CV47AL, UK

## Abstract

**Background:**

At least 3% of children spend some of their childhood in public care and, as a group, have poor outcomes across a range of education, employment, health and social care outcomes. Research, using social care or government datasets, has identified a number of risk factors associated with children entering public care but the utility of risk factors in clinical practice is not established. This paper uses routine primary health care data to see if risk factors for children entering public care can be identified in clinical practice.

**Methods:**

A nested case control methodology using routine primary care data from the United Kingdom. Health service use data were extracted for the 12 months before the case child entered public care and compared with 12 months of data for four control mother child pairs per case pair, matched on the age and sex of the child and the general practice. Exposures of interest were developed from a systematic review of the literature on risk factors associated with children entering public care.

**Results:**

Conditional logistic regression was used to investigate the combined effect of more than one exposure of interest. Maternal mental illness (OR 2.51, 95% CI 1.55-4.05), maternal age at birth of the child, socio-economic status (5^th ^quintile vs. 1^st ^quintile OR 7.14, 95% CI 2.92-17.4), maternal drug use (OR 28.8, 95% CI 2.29-363), non attendance at appointments (OR 2.42, 95% CI 1.42-4.14), child mental illness (OR 2.65, 95% CI 1.42-4.96) and child admission to hospital (OR 3.31, 95% CI 1.21-9.02) were all significantly associated with children entering public care. Maternal use of primary care contraception services was negatively associated with children entering public care (OR 0.52, 95% CI 0.31-0.87).

**Conclusions:**

Differences in health service use can be identified from routine primary care data in mother child pairs where children enter public care after controlling for maternal age and socio-economic status. The interaction between different risk factors needs testing in a cumulative risk model using longitudinal datasets.

## Background

Children in public care are a vulnerable group with '*a higher level of health, mental health and health promotion needs than others of the same age*' [1]. A large study of the mental health of children in public care in England showed a fivefold increase in mental disorder compared to other children, with conduct disorder contributing most of the difference in childhood psychopathology [2]. Elevated mental illness prevalence rates for children in public care are also reported from the USA [3,4], Australia [5] and Denmark [6]. Research shows that children in public care are under immunised [7] and providing information on immunisation status to social workers has not improved immunisation coverage [8]. In addition young people in public care have high levels of risk taking behaviours including smoking, alcohol and drug use [9-12] and are sexually active at an early age with high rates of teenage pregnancy recorded from the United Kingdom [13] and Sweden [14,15].

Policy and practice developments in the United Kingdom are leading to improvements in the health and wellbeing of children in public care [16,17] but, nevertheless, there is evidence from the UK and Sweden that young people in public care do less well than other young people across all five *Every Child Matters *[18] domains (be healthy, stay safe, enjoy and achieve, make a positive contribution and achieve economic wellbeing) [2,19-23].

At the start of a period of public care, one study found half of children had a diagnosable mental illness [24] and other research shows that they are more often involved in risk taking behaviours including early and unprotected sexual activity, smoking, alcohol and drug misuse than other young people of the same age [24-26]. Many younger children enter public care with developmental delay and a history of maltreatment including abuse and neglect which can have long term consequences.

These findings at entry to public care suggest that health issues are present and could be identified before entry to care; whilst children are living at home. To understand what is known of the health characteristics and social circumstances of families where children are subsequently taken into public care, a systematic review of risk factors associated with children entering public care was conducted [27]. This found that for mothers of children who enter public care, there is evidence of association with socioeconomic status [28-31], benefit receipt [28,29,32], single parenthood [28,31,32], ethnicity [28,31], age [20,21,29], disability [31], smoking in pregnancy [33], mental illness [29,33], alcohol misuse [34], sexually transmitted infections [35] and learning difficulties [29]. For children who enter public care, there is evidence of association with low birth weight and prematurity [33], disability [32], injuries [32], congenital syphilis [35] and attendance at Accident and Emergency departments [32]. However, much of the available information is derived from cohort studies of populations selected because families are already being in contact with social care services and that very little research has been carried out in the UK.

One important and previously unexplored source of information on risk factors leading up to a child entering public care is the data collected in primary health care, this has the advantage that it is routinely collected in practice, is almost universal in a UK context and could provide much more detailed health information than previous research on health associations for children entering public care. In addition, this dataset allows the health risk factors to be assessed alongside socio-economic status data, which may be confounding previously described health associations.

In this paper, we use primary health care data to explore the risk factors associated with children entering public care to address two research questions:

1. *Are risk factors associated with children entering public care identifiable from the health records of mothers of children who enter public care compared to mothers whose children do not enter public care?*

2. *Are risk factors associated with entering public care identifiable from the primary care health records of these children compared to children who do not enter public care?*

## Methods

### Ethical approval

In 2006, the Medical Research Council (MRC) began a scheme with the Medicines and Healthcare products Regulatory Agency (MHRA) to provide free access to GPRD data for up to 50 approved and academically led proposals per year. This project was awarded a licence as part of the MRC-MHRA collaboration and was approved by the Scientific and Ethical Advisory Group of GPRD as a suitable study.

We carried out a case control study; nested within a large primary care database: the General Practice Research Database (GPRD). We reviewed contemporaneous medical records of mother child pairs (dyads) where the child was recorded as having entered public care.

### Case and control definitions

We defined a case as a mother-child dyad, where both have been registered at the general practice and the child was recorded as having gone into public care. The period of time for which the primary care records are examined is a crucial decision. Too short a time period would risk missing key events, while too long would risk losing cases where the family were not registered with the primary care practice for long enough. We chose to analyse a period of 12 months before the child enters public care.

We chose to study the oldest child in the family when more than one sibling was taken into public care because this child is likely to have the most available health data. Because we required at least 12 months of data to be available, only children aged more than 1 year at entry to public care were included in the study. For each case dyad, staff at GPRD randomly selected four control dyads, matched by age and sex of the child and general practice.

### The General Practice Research Database

The GPRD is the largest database of anonymous longitudinal primary care information in the world. It was established in 1987, has 44 million patient years of high quality validated data, 3.6 million active patient records and 13 million records in total [36]. The database contains information on clinical events, coded by general practitioners at consultations, prescriptions, referrals to secondary care for appointments or investigations and information on admissions to hospital, all events are coded using Read/OXMIS codes, akin to International Classification of Disease codes for clinical diagnoses. There are Read/OXMIS codes identifying that a child has entered public care. The Townsend score [37], as a measure of a measure of socio-economic status (SES) based on post-codes, was available for patients living in part of England. Because we believed that socio-economic status was likely to be an important risk factor for entering care and a potential confounding factor for other associations previously described in the literature, we limited our analysis to case and control dyads where a Townsend score was available.

**Figure 1:**
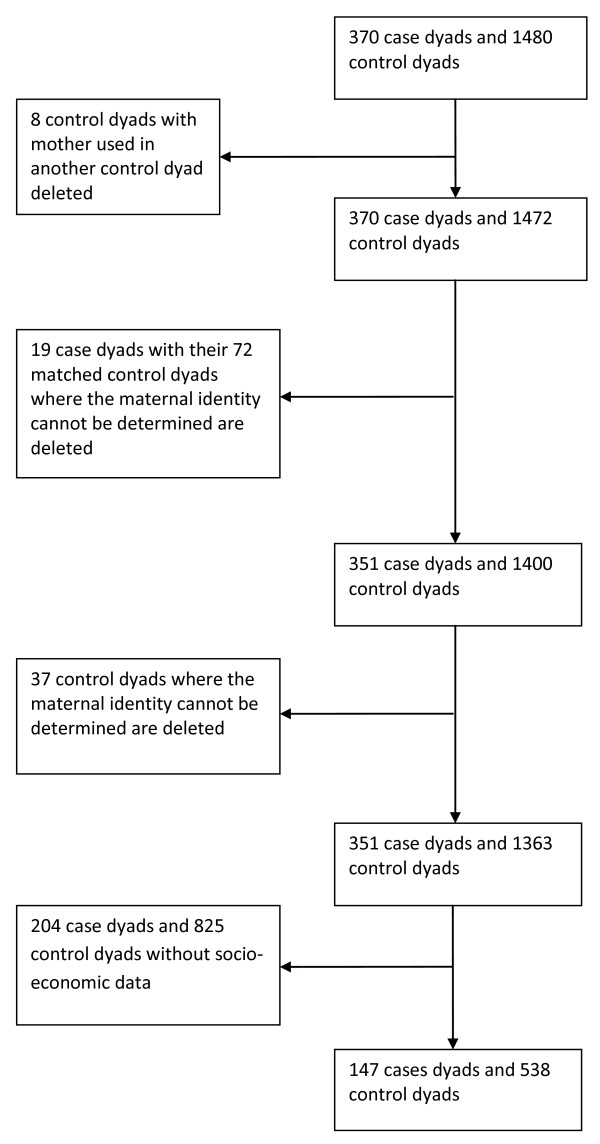
**Creating the final dataset**.

The dataset provided by GPRD consisted of 2,954 case dyads and 11,816 control dyads (4 × 2,954 = 11,816). The dataset needed to be refined as it included children less than I year old, data on siblings and dyads with less than 12 months of data available. The requirement for 12 months of up to research standard data was the step that most reduced the dataset. This narrowing of the dataset produced 370 case dyads and 1480 control dyads. Our requirement for socio-economic status data reduced the dataset further to 147 cases and 538 control dyads (Figure 1). We examined whether the dataset in which Townsend Score was available, differed from the larger dataset (370 case dyads and 1480 control dyads) by comparing the sex and age of the case children and maternal age and socio-economic status and found no statistical differences between the two datasets.

For this study a special link from mothers to their children was created by GPRD epidemiologists. In some cases there was more than one adult female (the GPRD mother-child link gave any adult female in a household who could, by date of birth, theoretically be the mother). We developed an algorithm using pregnancy related events in the adult females' files to identify the mother and where this was not possible we excluded the dyad. On eight occasions a control mother with two children was matched to two different case children, we excluded the control dyad with the younger child.

We converted all the relevant files into STATA files, and prepared a file for analysis including twelve months of medical records for case dyads that matched our inclusion criteria and four matched control dyads (matched on gender and age of the oldest child that was taken into care).

### Data validation

To validate our cases we sent a questionnaire to General Practitioners of a random sample of 100 cases to confirm that the case child was taken into public care. This was administered by GPRD to protect the identity of the practices and the families.

### Identifying variables for analysis

The systematic review of risk factors associated with children entering public care [27] and clinical experience identified a number of health risk factors to analyse in this primary care dataset. Because previous research has not described these risk factors in routine primary care data, we included all the previously recognised risk factors in our analysis. We included the variables socio-economic status and maternal age at birth of first child as these are well recognised risk factors for children entering public care and may also confound the association with health risk factors as both variables may influence health seeking behaviour.

The clinical exposures of interest were identified in the database from the clinical (Read/OXMIS) code used by the General Practitioner for a consultation, or from a relevant prescription. 'Sentinel events' were also coded; these were events or illnesses which may be associated with important social markers of vulnerability such as domestic violence. Non clinical exposures of interest in the mother files included maternal age at birth, socio-economic status, smoking, alcohol use, attendance at Accident and Emergency and referrals to secondary care that suggested a clinical exposure of interest. For the child, the non-clinical exposures included attendances at Accident and Emergency and referrals to secondary care that suggested a clinical exposure of interest.

Using codes identified from the clinical events, therapy and referral files we constructed 58 individual variables which were dichotomised to those individuals with the variable (at any frequency) and those without. Some variables were created by combination of other variables that relate to another aspect of the same issue, details of these composite variables are given in Table 1.

**Table 1:** Variables created by combining other variables

Title of composite variable	Created from individual variables
**Any social service input**	Social service input described in the maternal clinical events file Social service input in child clinical file
**Financial issues**	Financial issues in maternal clinical events file Financial issues in child clinical file
**Any nursing input**	Practice nurse involvement with mother in clinical events file Health visitor involvement recorded in maternal clinical events file District nurse involvement with mother in clinical events file Heath Visitor input in child clinical file, District or Practice nurse input in child clinical file
**Any non attendance at primary or secondary care**	Maternal non-attendance at primary or secondary care Maternal non attendance for cervical smear Any non attendance at primary or secondary care codes in child clinical file Immunisation data incomplete in child clinical file
**Child mental illness codes**	ADHD treatment in child prescription file Hypnotic in child prescription file Antipsychotic in child prescription file Referral to Child and Adolescent Mental Health Services in referral file Child mental illness codes in child clinical file Child behavioural issues in child clinical file, Antidepressant in child prescription file.
**Maternal mental illness codes**	Maternal depression in clinical events file Maternal prescription of an antidepressant Maternal bipolar disorder in clinical events file Maternal prescription for treatment of bipolar disorder Maternal referral to psychiatry Maternal mental illness in clinical events file Concern about maternal psychological health Maternal prescription of anxiolytic Maternal psychosis in clinical events file Maternal prescription of an antipsychotic medication
**Maternal alcohol misuse**	Maternal alcohol misuse Maternal prescription for treatment of alcohol misuse Sentinel conditions for alcohol misuse in maternal file
**Maternal drug misuse**	Maternal drug misuse in clinical events file Prescription of treatment for opiod misuse in maternal therapy file Sentinel conditions for drug misuse in maternal file
**Domestic abuse**	Domestic abuse in maternal clinical events file Sentinel conditions for trauma in maternal file
**Maternal depression**	Maternal depression in clinical events file Maternal prescription of an antidepressant
**Maternal bipolar disorder**	Maternal bipolar disorder in clinical events file Maternal prescription for treatment of bipolar disorder
**Maternal psychosis**	Maternal psychosis in clinical events file Maternal prescription of an antipsychotic medication
**Maternal contraception**	Maternal contraception in clinical events file Maternal prescription of contraceptive

### Statistical analysis

We explored the relationship between each variable and the risk of being taken into public care individually and in a stepwise multivariate conditional logistic regression model to investigate the combined effect of more than one exposure of interest. The variables child maltreatment and social service input (in both mother and child files) were not entered into the model as these correlated very highly with entering care and were not health service use issues. The variables with no odds ratio have a zero score on one of the 2 by 2 table fields and cannot be computed by this method, we assigned a p value for these variables using an exact form of McNemar's Test.

## Results

### Data validation

82 of the 100 questionnaires sent out by GPRD were returned by General Practices. 11 of the 82 simply said that they had no information on the child, usually because the child had transferred out of the practice. From the remaining 71 questionnaires, 66 (93%) confirmed that the child did enter public care. Four children may have been in foster care, and one questionnaire said that the child did not enter public care.

This suggests that approximately 7% of the dyads included as cases may not correctly be identified. The error in identification will attenuate any effect size identified in the study.

### Demographic information

**Figure 2:**
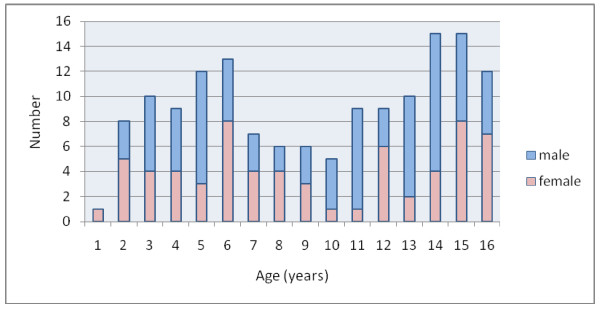
**Age and sex of case children**.

The age and gender of case children are described in Figure 2. Children in this study entered care more commonly under the age of six years or older than 14 years.

**Figure 3:**
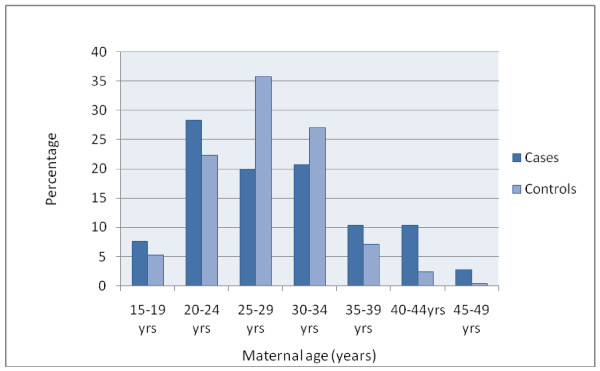
**Maternal age at time of birth**.

**Figure 4:**
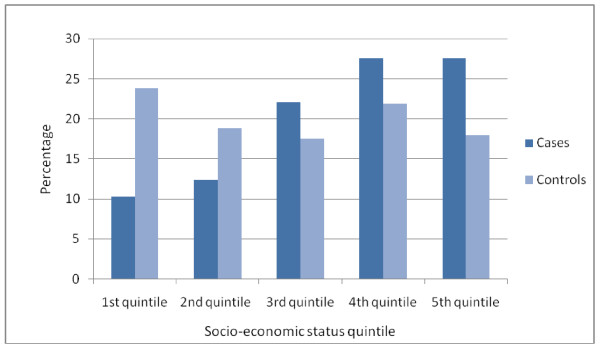
**Maternal socio-economic status quintile**.

The age of mothers at the birth of the index child shows a close approximation to a normal distribution for control mothers (Figure 3). However, there are more young case mothers than control mothers and there are more case mothers over the age of 35. Case mothers are also more likely to be in lower socio-economic quintiles (Figure 4).

Table 2 shows the results of individual variable conditional logistic regression while Table 3 shows the results of the stepwise conditional logistic regression model, created using all the variables significantly associated with children entering public care from Table 2. Maternal mental illness, maternal age at birth of the child, socio-economic status, maternal drug misuse, non attendance at appointments for mother or child, child mental illness and admission to hospital of the child were all significantly related to the risk of a child entering public care. Maternal use of primary care contraception services was negatively associated with children entering public care.

**Table 2:** Individual variables conditional logistic regression with the child entering public care

Exposure of interest	Cases n = 147	Controls n = 538	Odds Ratio (95% Confidence interval)	P value
Maternal mental illness	75 (51%)	132 (25%)	3.42 (2.29-5.11)	< 0.001
Maternal depression	52 (35%)	92 (17%)	2.76 (1.81-4.21)	< 0.001
Maternal referral to psychiatry	43 (28%)	58 (11%)	3.71 (2.29-6.01)	< 0.001
Maternal prescription of anxiolytic	29 (20%)	24 (4%)	5.49 (3.00-10.1)	< 0.001
Maternal psychosis	12 (8%)	6 (1%)	7.41 (2.78-19.8)	< 0.001
Concern about maternal psychological health	31 (21%)	55 (10%)	2.43 (1.46-4.04)	0.001
Maternal drug misuse	7 (5%)	1 (0.2%)	27.1 (3.33-220)	0.002
Maternal over dose referral	4 (3%)	0 (0%)	-	0.002*
Maternal alcohol misuse	6 (4%)	1 (0.2%)	23.1 (2.78-192)	0.004
Maternal visit to A + E	17 (12%)	29 (5%)	2.39 (1.27-4.52)	0.007
Maternal smoking	33 (22%)	76 (14%)	1.88 (1.17-3.03)	0.009
Maternal admission to hospital	6 (4%)	4 (0.7%)	6.42 (1.59-25.9)	0.009
Relationship issues in maternal file	5 (3%)	4 (0.7%)	4.69 (1.26-17.5)	0.021
Maternal contraception	41 (28%)	198 (37%)	0.64 (0.43-0.97)	0.035
Child mental illness	34 (23%)	43 (8%)	3.87 (2.29-6.53)	< 0.001
Child behavioural issues	19 (13%)	18 (3%)	4.80 (2.35-9.82)	< 0.001
Child maltreatment	20 (14%)	0 (0%)	-	< 0.001*
Child admission to hospital	12 (8%)	12 (2%)	3.83 (1.68-8.72)	0.001
Child referral to A + E	16 (11%)	25 (5%)	2.74 (1.37-5.52)	0.005
Any non attendance at primary or secondary care	57 (39%)	101 (19%)	2.95 (1.93-4.52)	< 0.001
Any social service input	31 (21%)	4 (0.6%)	55.8 (13.3-233)	< 0.001
Maternal age 15-19 years old	13 (9%)	28 (5%)	2.63 (1.24-5.59)	0.012
Maternal age 20-24 years old	41 (28%)	117 (22%)	2.34 (1.39-3.94)	0.001
Maternal age 25-29 years old	30 (20%)	197 (37%)	1.00	-
Maternal age 30-34 years old	30 (20%)	142 (26%)	1.35 (0.78-2.34)	0.282
Maternal age 35-39 years old	15 (10%)	39 (7%)	2.74 (1.33-5.67)	0.007
Maternal age 40-44 years old	14 (10%)	13 (2%)	6.97 (2.92-16.6)	< 0.001
Maternal age 45-49 years old	4 (3%)	2 (0.4%)	18.8 (1.64-158)	0.017
SES quintile 1	15 (10%)	131 (23%)	1.00	
SES quintile 2	18 (12%)	104 (19%)	1.71 (0.80-3.67)	0.163
SES quintile 3	32 (22%)	94 (17%)	3.38 (1.69-6.77)	0.001
SES quintile 4	41 (28%)	119 (22%)	3.92 (1.98-7.73)	< 0.001
SES quintile 5	41 (28%)	90 (17%)	6.51 (3.09-13.7)	< 0.001
Maternal smoking cessation	3 (2%)	8 (1%)	1.29 (0.34-4.89)	0.71
Maternal sexually transmitted infection	1 (0.7%)	19 (4%)	0.19 (0.03-1.47)	0.11
Maternal termination of pregnancy	2 (1%)	6 (1%)	1.28 (0.26-6.37)	0.760
Maternal referral to Orthopaedics	17 (11%)	38 (7%)	1.74 (0.95-3.20)	0.075
Sentinel conditions for maternal trauma	5 (3%)	14 (3%)	1.37 (0.49-3.88)	0.55
Domestic abuse	7 (5%)	18 (3%)	1.53 (0.62-3.75)	0.354
Maternal learning difficulties	1 (0.7%)	0 (0%)	-	0.2*
Maternal bipolar disorder	2 (1%)	1 (0.2%)	8.00 (0.73-88.2)	0.09
Sentinel conditions for maternal drug misuse	1 (0.7%)	1 (0.2%)	4.00 (0.25-64.0)	0.33
Sentinel conditions for alcohol misuse	1 (0.7%)	0 (0%)	-	0.2*
Child with scabies	0 (0%)	4 (0.7%)	-	0.409*
Child with head lice	9 (6%)	28 (5%)	1.35 (0.58-3.11)	0.485
Child health surveillance codes	13 (9%)	55 (10%)	0.79 (0.33-1.92)	0.604
Ethnicity in maternal file	0 (0%)	5 (1%)	-	0.328*
Child developmental delay	4 (3%)	12 (2%)	1.29 (0.39-4.21)	0.678
Child immunisation data incomplete	1 (0.7%)	2 (0.4%)	2.45 (0.14-42.6)	0.539
Immunisation refused	2 (1%)	1 (0.2%)	7.29 (0.66-80.8)	0.105
Child referrals to orthopaedics	1 (0.7%)	7 (1%)	0.52 (0.06-4.31)	0.551
Other child referrals to secondary care	18 (12%)	54 (10%)	1.27 (0.70-2.29)	0.428
Sentinel conditions for child trauma	3 (2%)	8 (1%)	1.26 (0.33-4.77)	0.738
Sentinel conditions for child head injury	2 (1%)	11 (2%)	0.68 (0.15-3.16)	0.627
Sentinel codes for child burn	3 (2%)	2 (0.4%)	5.36 (0.89-32.2)	0.067
Child smoking	2 (1%)	4 (0.7%)	2.93 (0.35-10.4)	0.458
Child contraception	4 (3%)	7 (1%)	2.93 (0.62-13.9)	0.174
Child pregnancy	2 (1%)	4 (0.7%)	4.45 (0.32-62.7)	0.269
Relationship issues in child file	1 (0.7%)	1 (0.2%)	4.00 (0.25-64.0)	0.333
Antidepressant prescribed to child	3 (2%)	3 (0.6%)	5.04 (0.82-31.0)	0.081
Hypnotic prescribed to child	8 (5%)	19 (4%)	1.63 (0.68-3.91)	0.271
Child ADHD treatment	3 (2%)	3 (0.6%)	3.40 (0.68-17.1)	0.137
Child alcohol concern	1 (0.7%)	2 (0.4%)	2.00 (0.18-22.1)	0.571
Child drug misuse	1 (0.7%)	0 (0%)	-	0.2*
Health Visitor involvement	4 (3%)	5 (1%)	8.64 (0.90-83.0)	0.062
Any nursing input	4 (3%)	8 (1%)	2.36 (0.56-10.0)	0.243
Financial issues	3 (2%)	5(1%)	2.23 (0.53-9.35)	0.273

The variables with no odds ratio have a zero score on one of the 2 by 2 table fields and cannot be computed by this method, we assigned a p value for these variables using an exact form of McNemar's Test

**Table 3:** Forward selection conditional logistic regression model

Exposure of interest	Cases n = 147	Controls n = 538	Odds ratio (95% confidence intervals	P value
Maternal mental illness	75 (51%)	132 (25%)	2.51 (1.55-4.05)	< 0.001
Maternal age 15-19 years old	31 (21%)	4 (0.6%)	2.45 (1.11-5.42)	0.027
Maternal age 20-24 years old	13 (9%)	28 (5%)	2.37 (1.38-4.06)	0.002
Maternal age 25-29 years old	41 (28%)	117 (22%)	1.00	0.236
Maternal age 30-34 years old	30 (20%)	197 (37%)	1.43 (0.81-2.53)	0.222
Maternal age 35-39 years old	30 (20%)	142 (26%)	2.90 (1.36-6.18)	0.006
Maternal age 40-44 years old	15 (10%)	39 (7%)	6.21 (2.50-15.4)	< 0.001
Maternal age 45-49 years old	14 (10%)	13 (2%)	24.4 (2.30-258)	0.008
SES quintile 1	15 (10%)	131 (23%)	1.00	-
SES quintile 2	18 (12%)	104 (19%)	2.41 (1.01-5.74)	0.047
SES quintile 3	32 (22%)	94 (17%)	2.83 (1.25-6.39)	0.012
SES quintile 4	41 (28%)	119 (22%)	3.40 (1.58-7.32)	0.002
SES quintile 5	41 (28%)	90 (17%)	7.14 (2.92-17.4)	< 0.001
Maternal drug misuse	7 (5%)	1 (0.2%)	28.8 (2.29-363)	0.009
Any non attendance at primary or secondary care	57 (39%)	101 (19%)	2.42 (1.42-4.14)	0.001
Child mental illness	34 (23%)	43 (8%)	2.65 (1.42-4.96)	0.002
Maternal contraception	41 (28%)	198 (37%)	0.52 (0.31-0.87)	0.013
Child admission to hospital	12 (8%)	12 (2%)	3.31 (1.21-9.02)	0.019

## Discussion

Using a primary care database is a novel approach to identifying risk factors for children entering public care. Most of published literature has studied families already in contact with social care services, but such families are a minority of the population, already identified as having a problem of some kind. By contrast, primary care databases include an unselected proportion of the whole population registered with the health service, giving a new perspective on the events leading up to a child being taken into care. A very high percentage of families in the UK are registered with a general practice so identifiable risk factors in routine primary care data could allow access to interventions at a much earlier stage than identification via contact with social care services.

We defined a case as a mother child dyad, where the child was the oldest child in the family and both mother and child had 12 months of up to research standard data available. The 12 months requirement was a judgement based on an expectation that most significant health issues would be recorded in the database over that time period. However, excluding children with less than 12 months data considerably reduced the number of included cases. A shorter timeframe would have included more cases and increased the power of the study. It may also have included some highly mobile families that do not stay with the same general practice for 12 months, and research [30,32] suggests that families that are highly mobile have a higher likelihood of children entering public care. However a shorter time frame increases the risk that relevant health issues are not recorded in the primary care record and we considered 12 months to be an appropriate balance between completeness of data and the number of included dyads.

The individual variable results presented in Table 2 shows twenty three variables were significantly associated with children entering public care on univariate analysis, but only eight remained significant in the stepwise multivariate conditional logistic regression model. A striking finding is that individual variables may be highly specific but relatively insensitive. Maternal alcohol misuse is an example; this was described in the 6 case mothers and 1 control mother making the association statistically significant as an individual variable. Other epidemiological evidence suggests that alcohol misuse is under-recorded in the primary care record; the National Institute for Health and Clinical Excellence makes a recommendation on screening for alcohol consumption in primary care in the 2010 guidance on preventing hazardous and harmful drinking [38] and it is possible that some alcohol misuse does not appear in the primary care dataset because general practitioners do not systematically screen for it.

### Maternal mental illness

One of our most important findings is the association of maternal mental illness with children entering public care. Other research has identified an association of parental mental illness with children entering public care, but the definitions of mental illness vary considerably between studies and differ from that used in this research. Franzén et al. used admission to hospital for psychiatric illness, attempted suicide or substance misuse for either parent to create a 'psychosocial risk' variable [29]. This identifies more serious mental illness than we will have identified from primary care records and a level of illness where the possibility of early intervention may already have passed.

Some adult mental health services recognise that their patients may also be parents and that parental mental illness may impact upon parenting [39]. The issue has been highlighted in a study of women with psychotic disorders in south London [40]. 155 (63%) of these women with psychotic disorders were also mothers but only sixteen of them had a history of having a child enter public care, meaning that 90% of these women were caring for their own children [40]. The fear of children entering public care is felt especially in the context of episodes of in-patient care and although not the only reason why parents with mental illness are reluctant to ask for help with parenting, it may be the most pervasive. People with mental illness face many challenges including poverty, emotional distress, cognitive impairment, medication side effects and perceived stigma, so perhaps it is unsurprising that few people with mental illness seek or receive specific help with parenting. There is a contrast with the resources dedicated to helping these patients with other issues such as securing benefits, finding accommodation, maintaining gainful occupation and adhering to medication [41].

The association of maternal mental with children entering public care is a potentially important finding of this study with implications for adult psychiatry and primary care services. However, it is possible that the observed relationship between an increased risk of children going into care and maternal mental illness is due to confounding by some other variable. For example, Diaz-Caneja and Johnson reported that mothers who felt it was probable that their children would be taken into care became concerned, anxious or depressed and sought mental health support [42]. Further research in large cohorts may help to disentangle these factors.

### Maternal age at birth of child

Both younger and older mothers were significantly more likely to have their children enter public care, although the absolute number of older mothers was small. A number of previous studies have identified young mothers as a group with an increased risk of children entering public care [29,31] and two studies identified older mothers at increased risk also [30,33]. Of course teenage motherhood is not necessarily bad [43] and most pregnant teenagers do not have children that enter public care. However young motherhood has been associated with a range of negative outcomes in the USA and the UK, and both counties target this group of mothers for interventions [44,45].

### Socio-economic status

Low socio-economic status has been recognised as an important risk factor associated with children entering public care in many studies. In the UK context, the work of Bebbington and Miles [28] has been influential in social care and government guidance and policy development [46,47]. Their findings were replicated in Sweden by Franzén, Vinnerljung and Hjern [29] and it is clear that this is an important risk factor. Figure 4 shows that, while control families were approximately equally distributed across the quintiles of the Townsend score, case families showed an increase from the 1^st ^to 4^th ^quintiles and remained high in the 5^th ^(most disadvantaged) quintile.

The association of material deprivation with many indices of poor health has been frequently reported in the United Kingdom [48-53] and strategies to reduce social inequality remain necessary. There has been vigorous debate on the mechanisms, models and causal pathways by which material deprivation exerts its negative impact on a wide range of child health outcomes. As many people experience multiple disadvantage and deprivation, Spencer suggests that single causal factors are of limited value and theoretical models which accommodate the cumulative effects of various factors over time are more likely to offer a fruitful approach to explaining the relationship between socio-economic status and health [50].

The relationship between socio-economic status and health service use is an important issue. It could be that low socio-economic status is the prime driver of children entering public care, and patterns of health service use simply reflect the consequences of deprivation. However Franzén found a significant association between psychosocial markers and children entering care after adjusting for socio-economic status [29] and in this study variables for health service use have a significant relationship with risk of a child entering care after adjusting for socio-economic status (Table 3).

A plausible scenario is that there are several routes that lead to children entering public care and the risk factors we have identified are all pathways to children entering public care. It is possible that the stresses are additive with an aggregate of stressors leading to children entering public care [54,55].

### Other maternal factors

Eight mothers of the mothers in this study were recorded as having a drug misuse problem; seven of them had children who entered public care. In the USA, parental problem drug use is one of the main reasons for children entering public care [56]. The Advisory Council on the Misuse of Drugs reported that 2-3% of all children under the age of 16 have parents with drug problems in the UK [57], which suggests that we have not identified some mothers with drug problems, maybe because most drug use is not recorded in primary care. Percy et al. call for more recognition of the needs of dependent children within adult treatment services working with parents [58]. This will probably require much closer working between addiction services and primary care.

Maternal non-attendance at appointments and failure to use contraceptive services were both significantly related to a higher risk of children being taken into care. These variables may be indicators of a chaotic lifestyle. Successful engagement with the National Health Service requires some planning and non-attendance at appointments is common at secondary care out-patient clinics, varying between 5 and 34%, but is more infrequent in primary care at 3-6.5% [59,60]. Non attendance is a recurring theme in serious case reviews of child maltreatment and many NHS organisations have now developed non attendance policies.

### Child related factors

The children who entered public care were more than 2.5 times as likely to have a mental illness in the preceding 12 months as control children. Children in difficult life circumstances may express their unhappiness in ways that result in presentation to health services. Experiences of abuse are associated with mental ill health in children, and a recent systematic review demonstrated that the strongest associations between parent-child relationships and psychiatric disorder in adult offspring were for the most severe forms of physical and sexual abuse, and neglect in childhood [61]. An Australian study of the mental health of 347 children in public care found that the strongest pre-care predictor of children's mental ill-health was 'age at entry to care', an indicator of overall exposure to pre-care adversity [62]. Mental illness could be reduced if services can identify these children earlier, but it is also important to avoid false positive interventions that result in children being improperly placed in public care.

Case children were more than three times as likely as control children to have been in hospital during the 12 month period, although admission was an unusual event. Only 8% of case and 2% of control children were hospitalised. It may be that it is the admission itself that identifies problems in the family for the first time and leads to social care input.

## Conclusion

Research, using social care or government datasets, has identified risk factors associated with children entering public care but the utility of risk factors in clinical practice is not established. Using primary care data from the General Practice Research Database, this study identifies differences in health service use in mother child pairs where children enter public care after controlling for maternal age and socio-economic status. Maternal mental illness, maternal drug use, non attendance at appointments, child mental illness and child admission to hospital were all significantly associated with children entering public care and thus populations can be identified that could be targeted for secondary prevention strategies.

Practitioners, particularly general practitioners and adult mental health workers need to recognise that their patients may also be mothers and their illnesses may impact on their ability to parent; As Howard said '*parenting is not generally considered to be a mental health issue unless child protection concerns are raised'*[63].

Primary care practitioners need to screen for and record to record risk factors as it is likely that there is under reporting of risk factors in the clinical record.

## Competing interests

The authors declare that they have no competing interests.

## Authors' contributions

All the authors of this paper have made a substantial contribution to the conception and design, or analysis and interpretation of data, each has been involved in drafting the article or revising it critically for important intellectual content and each has given final approval of this version to be published.

## Sponsor statement

This study is based in part on data from the Full Feature General Practice Research Database obtained under licence from the UK Medicines and Healthcare Products Regulatory Agency (MHRA). However, the interpretation and conclusions contained in this study are those of the authors alone. Access to the GPRD database was funded through the Medical Research Council's licence agreement with MHRA.

Dr D E Simkiss is the guarantor for this manuscript.

## Pre-publication history

The pre-publication history for this paper can be accessed here:

http://www.biomedcentral.com/1472-6963/12/65/prepub
